# Pemphigus Foliaceus Mimicking Ichthyosis: A Rare Clinical Presentation

**DOI:** 10.7759/cureus.74675

**Published:** 2024-11-28

**Authors:** Adnan Ahmad, Fatmah Al-Sughayer, Manaf Shaban, Shatha Al-Roomi

**Affiliations:** 1 Dermatology, Ministry of Health, Sulibekhat, KWT; 2 Dermatology, Kuwait Institute for Medical Specializations, Sulibekhat, KWT; 3 Family Medicine, Ministry of Health, Sulibekhat, KWT; 4 Dermatology, Amiri Hospital, Ministry of Health, Kuwait City, KWT

**Keywords:** blistering disease, ichthyosis, pemphigus foliaceus, pemphigus vulgaris, plaque, scale

## Abstract

Pemphigus foliaceus (PF) is an autoimmune blistering disease characterized by the disruption of the epidermal cell adhesion protein desmoglein 1 (DsG1). PF classically presents with superficial erosions or blisters, but can rarely mimic other dermatological conditions, which makes diagnosis challenging. We report the case of a 57-year-old Sri Lankan man with a one-month history of widespread ichthyosis-like plaques and scales which started on his scalp and progressed in a cranio-caudal fashion and were associated with pruritus and few blisters. Histopathology revealed features consistent with PF, and immunofluorescence studies further confirmed the diagnosis of PF. This case highlights the atypical presentation of PF resembling ichthyosis, emphasizing the need to consider autoimmune blistering diseases for unexplained scaling dermatoses. The unusual nature of PF presenting with ichthyosis-like scaly plaques highlights the need for further research. Accurate early diagnosis and treatment can both significantly improve outcomes.

## Introduction

Pemphigus foliaceus (PF) is an autoimmune blistering disease characterized by superficial blisters due to the autoantibody-mediated disruption of the epidermal cell adhesion protein desmoglein 1 (DsG1) and not desmoglein 3 (DsG3) [1]. If both DsG1 and DsG3 antibodies were positive, then it is pemphigus vulgaris (PV) and not PF [1,2]. PF tends to mainly involve the superficial layers of the epidermis and typically presents with erosions or blisters without mucosal involvement [3]. PF rarely manifests with crusted erosions, erythema, and scaling, which can mimic other dermatoses such as psoriasis (PsO) and ichthyosis creating a diagnostic challenge [4]. A study that was conducted in South America mentioned the presence of PF cases mimicking ichthyosis [5]. We present the case of a 57-year-old Sri Lankan man with a unique presentation of PF that closely resembled ichthyosis.

## Case presentation

**Figure 1:**
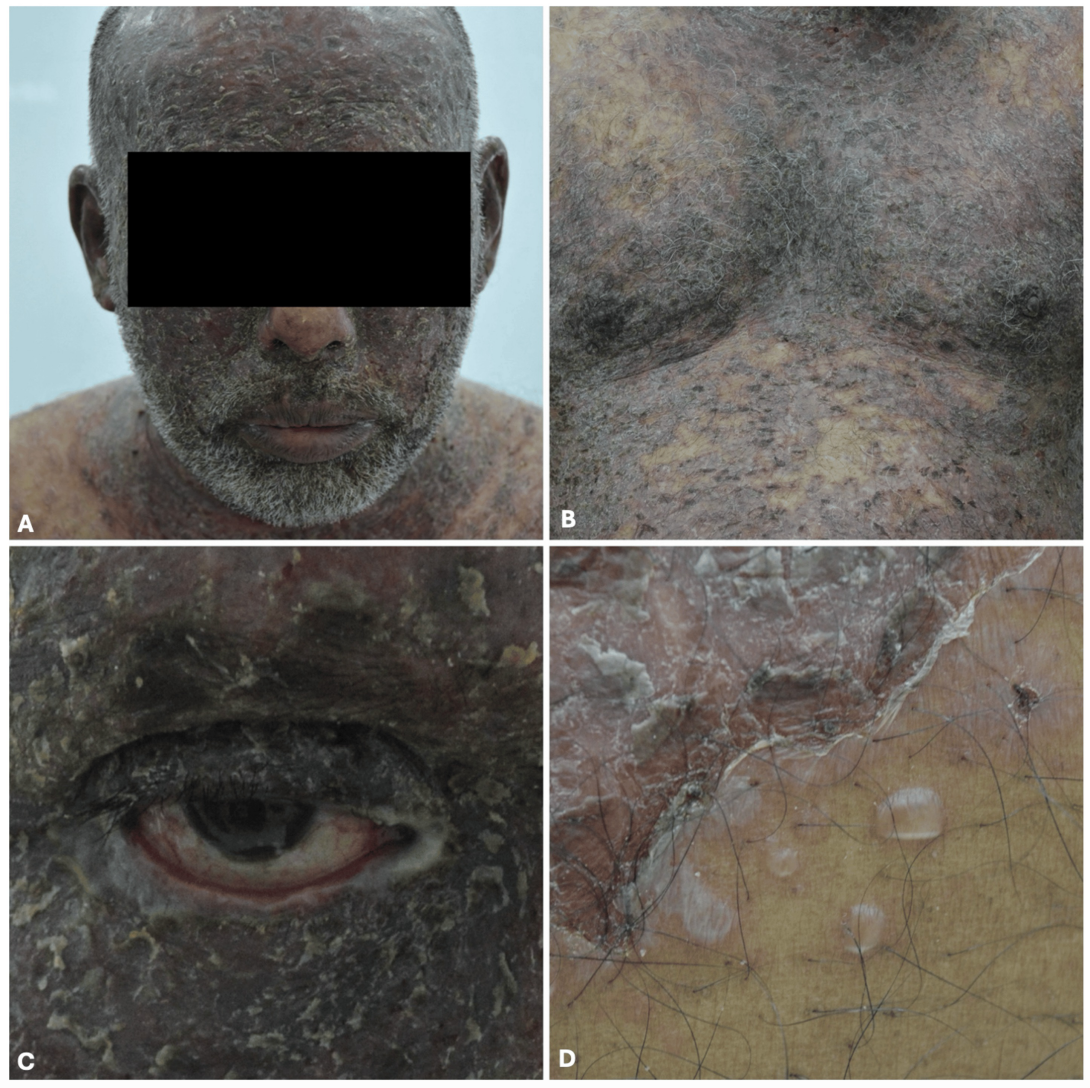
(A) Multiple scaly ichthyosis-like plaques were seen on the face. (B) Chest examination showed multiple scaly ichthyosis-like plaques. (C) Ectropion of the right eye was noted which was also present on the left eye. (D) Flaccid bullae were seen on the right side of the abdomen adjacent to scaly ichthyosis-like plaques.

We herein report the case of a 57-year-old Sri Lankan man who presented to our dermatology department with a one-month history of ichthyosis-like skin lesions distributed all over his body but sparing his groin, gluteal areas, bilateral legs, palms, and feet. The lesions started on his scalp and gradually spread in a cranio-caudal fashion and were itchy. The patient has no past medical history of chronic illnesses and this is his first episode. He has no known medical allergies and he does not take any medications. He works as a chauffeur for a family and he denied any sick contacts or recent infections. He also denied a history of alcohol consumption, illicit substance abuse, or smoking. Initially, he sought the opinion of different physicians but no accurate diagnosis was obtained. Physical examination revealed multiple scaly ichthyosis-like plaques on the skin distributed over his scalp, face, neck, shoulders, upper limbs, chest, abdomen, back, upper buttocks, and bilateral thighs (see Figure 1A, 1B). Ectropion of the eyes was also noted (see Figure 1C). Moreover, flaccid bullae were noted on the right side of his abdomen and waist as well as his right upper buttock (see Figure 1D). Furthermore, his oral mucosa and examination of the nails and genitalia were unremarkable.

**Figure 2:**
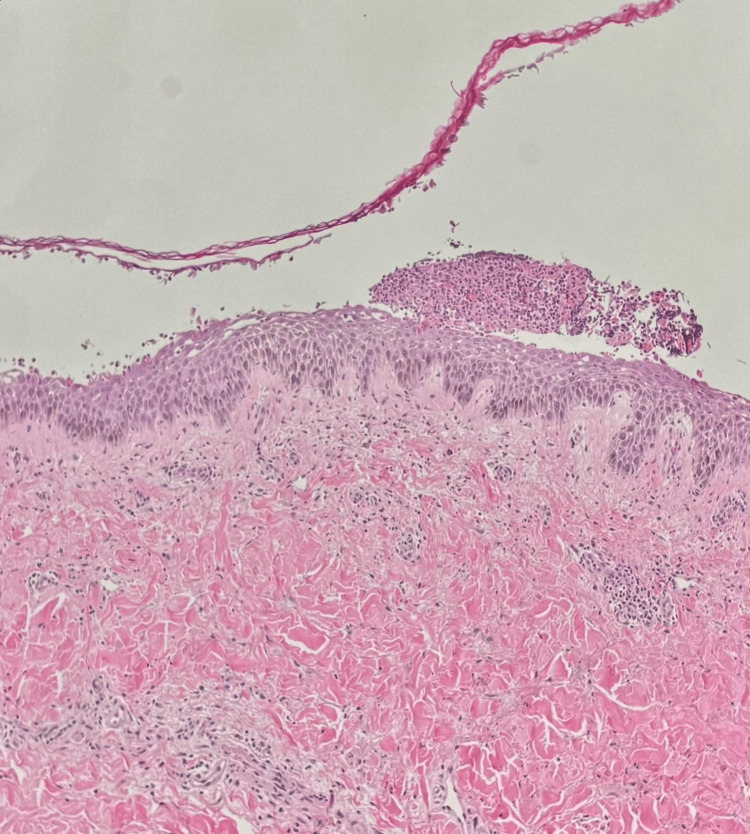
Acantholysis, detached stratum corneum, and a neutrophilic cell infiltrate in the superficial layers of the epidermis were observed with the upper dermis showing subcorneal blisters, mild perivascular lymphocytic cell infiltrate, and few eosinophils using H&E stain. H&E: hematoxylin and eosin

**Figure 3:**
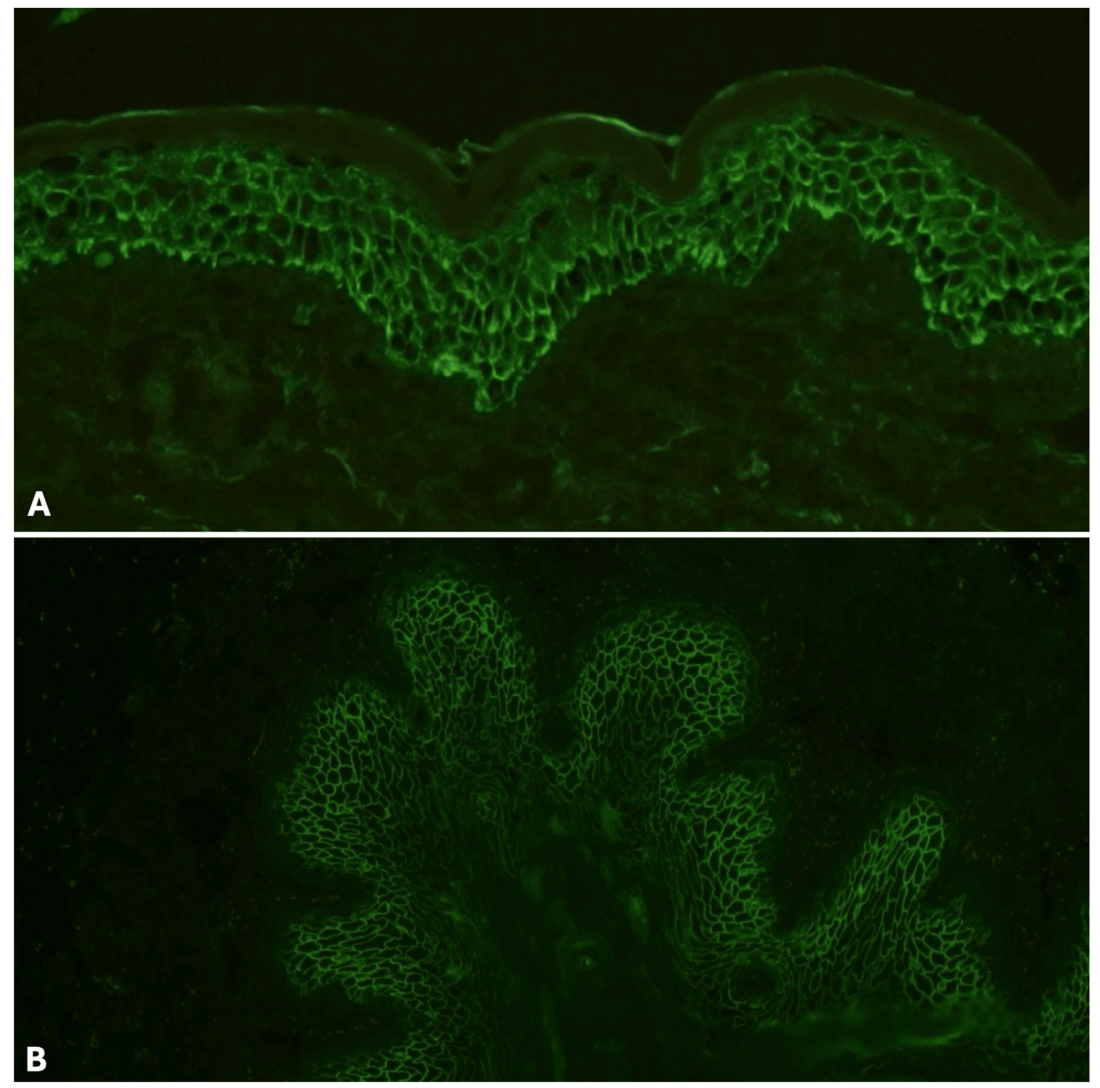
(A) DIF revealed fluorescent epidermal intercellular staining of IgG and C3. (B) IIF of ME showed positive staining of intercellular deposition. DIF: direct immunofluorescence; IIF: indirect immunofluorescence; ME: monkey esophagus

Our main differential diagnoses were impending erythroderma, bullous pemphigoid (BP), PV, acquired ichthyosis, plaque PsO, and pustular PsO. Under local anesthesia, we took both lesional and perilesional punch biopsies for histopathology and immunofluorescence studies, respectively. Furthermore, we prescribed oral prednisolone 60 mg once daily, and the patient started to gradually improve. Histopathological examination using hematoxylin and eosin (H&E) stain revealed features suggesting PF including subcorneal blisters with acantholysis, detached stratum corneum, and a neutrophilic cell infiltrate in the superficial layers of the epidermis. The upper dermis showed mild perivascular lymphocytic cell infiltrate with few eosinophils (see Figure 2). Direct immunofluorescence (DIF) test showed fluorescent epidermal intercellular staining of IgG and C3 (see Figure 3A). Indirect immunofluorescence (IIF) test of monkey esophagus (ME) also revealed positive staining of intercellular deposition (see Figure 3B). Finally, enzyme-linked immunosorbent assay (ELISA) showed a high titer of DsG1 autoantibodies measuring 184.7 which was considered positive since it was above 36. On the other hand, the titer of DsG3 autoantibodies was 17.2 which was considered negative since it was below 19. Hence, our final diagnosis was PF.

## Discussion

PF classically presents with superficial blisters and erosions distributed over the scalp, face, shoulders, and upper trunk [1]. However, scaly plaques resembling ichthyosis are unusual for PF [5]. Moreover, a case report from the literature mentioned a rare variant of PF presenting with erythroderma resembling our case [6]. Ichthyosis is typically characterized by generalized scaling, often due to inherited or systemic diseases rather than autoimmune [7]. Acquired ichthyosis, while rare, is typically associated with systemic diseases such as malignancies, infections, or metabolic disorders, which were absent in our patient [8]. Given this unusual presentation, our initial differential diagnoses included impending erythroderma, BP, PV, acquired ichthyosis, plaque PsO, and pustular PsO. Ultimately, histopathological examination and immunological studies proved essential in differentiating PF from other diagnoses. Based on the literature, histopathological features of PF include characteristic subcorneal acantholysis and neutrophilic infiltrates [9].

Moreover, immunofluorescence studies further solidify the diagnosis, with DIF showing epidermal intercellular staining of IgG and C3, a hallmark of PF, and IIF of ME confirming the presence of IgG autoantibodies from the serum [9]. High levels of DsG1 autoantibodies, detected through ELISA, helped differentiate between PF and PV, as DsG3 autoantibodies were negative in our patient [9]. The significance of this case lies in the rarity of PF presenting in a form closely mimicking ichthyosis, which may lead to confusion, misdiagnosis, and delay in appropriate management. Therefore, early and accurate diagnosis can reduce morbidity and improve outcomes. Our case highlights the importance of considering PF in patients with unexplained ichthyosis-like lesions, particularly when typical ichthyotic patterns are absent or when ichthyotic lesions are associated with pruritus and blisters. Our case contributes to the limited literature on atypical PF cases and may help guide clinicians in recognizing and diagnosing similar unusual cases in the future.

## Conclusions

The case of PF, which resembled ichthyosis, highlights the complexities and diagnostic challenges associated with atypical autoimmune blistering diseases. PF classically presents with superficial blisters and erosions, but the patient's ichthyosis-like plaques led to the consideration of other diagnoses such as PsO and acquired ichthyosis. This case emphasizes the importance of thorough immunopathological evaluation to differentiate PF from other dermatoses. The report advocates for increased clinical awareness and consideration of autoimmune etiologies in cases of unusual scaling, especially when classic ichthyotic-like patterns and systemic associations are absent. Accurate and timely diagnosis is crucial for appropriate treatment and improved patient outcomes.
